# Experimental Evaluation of the Heat Sink Effect in Hepatic Microwave Ablation

**DOI:** 10.1371/journal.pone.0134301

**Published:** 2015-07-29

**Authors:** Kristina I. Ringe, Carolin Lutat, Christian Rieder, Andrea Schenk, Frank Wacker, Hans-Juergen Raatschen

**Affiliations:** 1 Department of Diagnostic and Interventional Radiology, Hannover Medical School, Hannover, Germany; 2 Institute for Medical Image Computing, Fraunhofer MEVIS, Bremen, Germany; National Yang-Ming University, TAIWAN

## Abstract

**Purpose:**

To demonstrate and quantify the heat sink effect in hepatic microwave ablation (MWA) in a standardized ex vivo model, and to analyze the influence of vessel distance and blood flow on lesion volume and shape.

**Materials and Methods:**

108 ex vivo MWA procedures were performed in freshly harvested pig livers. Antennas were inserted parallel to non-perfused and perfused (700,1400 ml/min) glass tubes (diameter 5mm) at different distances (10, 15, 20mm). Ablation zones (radius, area) were analyzed and compared (Kruskal-Wallis Test, Dunn’s multiple comparison Test). Temperature changes adjacent to the tubes were measured throughout the ablation cycle.

**Results:**

Maximum temperature decreased significantly with increasing flow and distance (p<0.05). Compared to non-perfused tubes, ablation zones were significantly deformed by perfused tubes within 15mm distance to the antenna (p<0.05). At a flow rate of 700ml/min ablation zone radius was reduced to 37.2% and 80.1% at 10 and 15mm tube distance, respectively; ablation zone area was reduced to 50.5% and 89.7%, respectively.

**Conclusion:**

Significant changes of ablation zones were demonstrated in a pig liver model. Considerable heat sink effect was observed within a diameter of 15mm around simulated vessels, dependent on flow rate. This has to be taken into account when ablating liver lesions close to vessels.

## Introduction

Percutaneous thermoablation by means of radiofrequency (RFA) or microwave ablation (MWA) are accepted and well established minimally invasive treatment options for primary and metastatic hepatic malignancies. The goal of these ablation procedures is to eradicate all viable malignant cells within a designated target volume by heating the tissue to temperatures in which irreversible injury occurs (e.g. 50–54°C for 4–6 minutes [1]. In this context, the extent of coagulation necrosis is dependent not only on the amount of energy deposited, but also on local tissue interactions and heat loss [2]. Blood vessels (diameter ≥ 3mm) abutting the target lesion prevent large temperature variations in the part of the tumor close to the vessel, thereby keeping the tissue cooler [3]. This so-called heat sink effect limits the effectiveness of all thermal ablation methods resulting in incomplete ablation and local tumor recurrence [4].

In the context of RFA, the heat sink effect has been investigated thoroughly both, in vitro and in vivo [5–7]. With MWA there is some evidence in the literature that the vascular cooling effect may be less relevant. Possible advantages of MWA include higher attainable temperatures in the target tissue based on an improved convection profile and a larger zone of active heating [8, 9]. While some authors did not find a heat sink effect with MWA [10, 11], other studies describe cooling effects less pronounced than with RFA [12–14].

Concisely, systematic evidence concerning the degree of vessel cooling in MWA with reference to contributing factors is very limited. The purpose of this study is to demonstrate and quantify the heat sink effect in microwave ablation of liver tissue in a standardized ex vivo liver model, and to analyze the influence of vessel distance and blood flow on lesion volume and shape.

## Materials and Methods

### Ablation procedure

This study was exempt from approval by our Institutional Animal Care and Use Committee. 108 ex vivo microwave ablation procedures were performed in pig livers, which were obtained from a slaughterhouse (Fa. Vasterling, Hannover, Germany) and used within 5 hours after harvesting. Livers were cut into smaller pieces suitable for the ablation procedure. MWA (10 minute cycle) was performed at room temperature using the Evident Microwave Ablation System (Covidien, Boulder, CO, USA), which consisted of a generator with a maximum power of 45W coupled to an internally cooled antenna with an active tip of 37mm. Antennas were inserted at different distances (10, 15 and 20mm) parallel to glass tubes (diameter 5mm), simulating hepatic vessels. For exact placement of microwave antennas and glass tubes a custom-made aiming device made of polymethyl methacrylate (PMMA) was used. The glass tube, in the following vessel, was connected to a roller pump with adjustable flow rate and perfused with 0, 700 and 1400 ml/min of saline at room temperature. Flow rates were chosen to reflect portal venous and total hepatic blood flow, respectively [15]. For each combination of vessel distance and perfusion rate 12 ablation procedures were performed. In addition, temperature adjacent to the glass tubes (facing the MWA antenna) was measured continuously throughout the ablation cycle until three minutes after end of the procedure using a radiofrequency ablation (RF) probe (Valleylab CoolTiP RF Ablation System, Valleylab Inc., Boulder, CO, USA), which was placed using the custom-made aiming device as well.

### Image analysis

Image analysis was performed by one radiologist experienced in performing ablation procedures and assessing post-interventional imaging in clinical routine (<10 years), using dedicated software (MeVis RF-Lesion Tool, Fraunhofer MeVis, Bremen, Germany), which facilitates measurement of the resulting zone of ablation. The radiologist was blinded to the condition of each experiment. This approach has been described in detail by Lehmann et al. for assessment of RFA lesions [16]. In brief, after the ablation procedure the liver was cut in half perpendicular at the center of the antenna and photographed. Images were transferred to the software tool for further analysis. The border of the ablation zone was determined and drawn manually based on the macroscopically detectable color change of the coagulated liver tissue [17]. Further, the positions of the microwave antenna and of the simulated vessel were marked. The software automatically calculated the minimum (r_min_) and maximum radius (r_max_), as well as the total area of the ablation zone. In addition, the idealized ablation zone area as a measure of a circular zone without cooling effects was calculated based on the maximum radius (idealized ablation zone = πr_max_^2^).

### Statistical analysis

Statistical analysis was performed using GraphPad Prism Software (version 6; GraphPad Software, Inc., USA). Ablation zone characteristics (r_min_, r_max_, total ablation zone area, idealized ablation zone area) and maximum temperature between perfused and non-perfused vessels based on different vessel distances were compared. For comparisons between two independent groups the Kruskal-Wallis Test was applied; for comparison between more independent groups Dunn’s multiple comparison Test was performed. For all statistical analyses p<0.05 was deemed significant.

## Results

### Morphology of the ablation zone

In non-perfused vessels, there was no significant difference of ablation zone characteristics (r_min_, r_max_, total ablation zone area, idealized ablation zone area) with regards to different distances between microwave antenna and vessel (p = 0.41, 0.38, 0.58 and 0.38, respectively).

**Fig 1:**
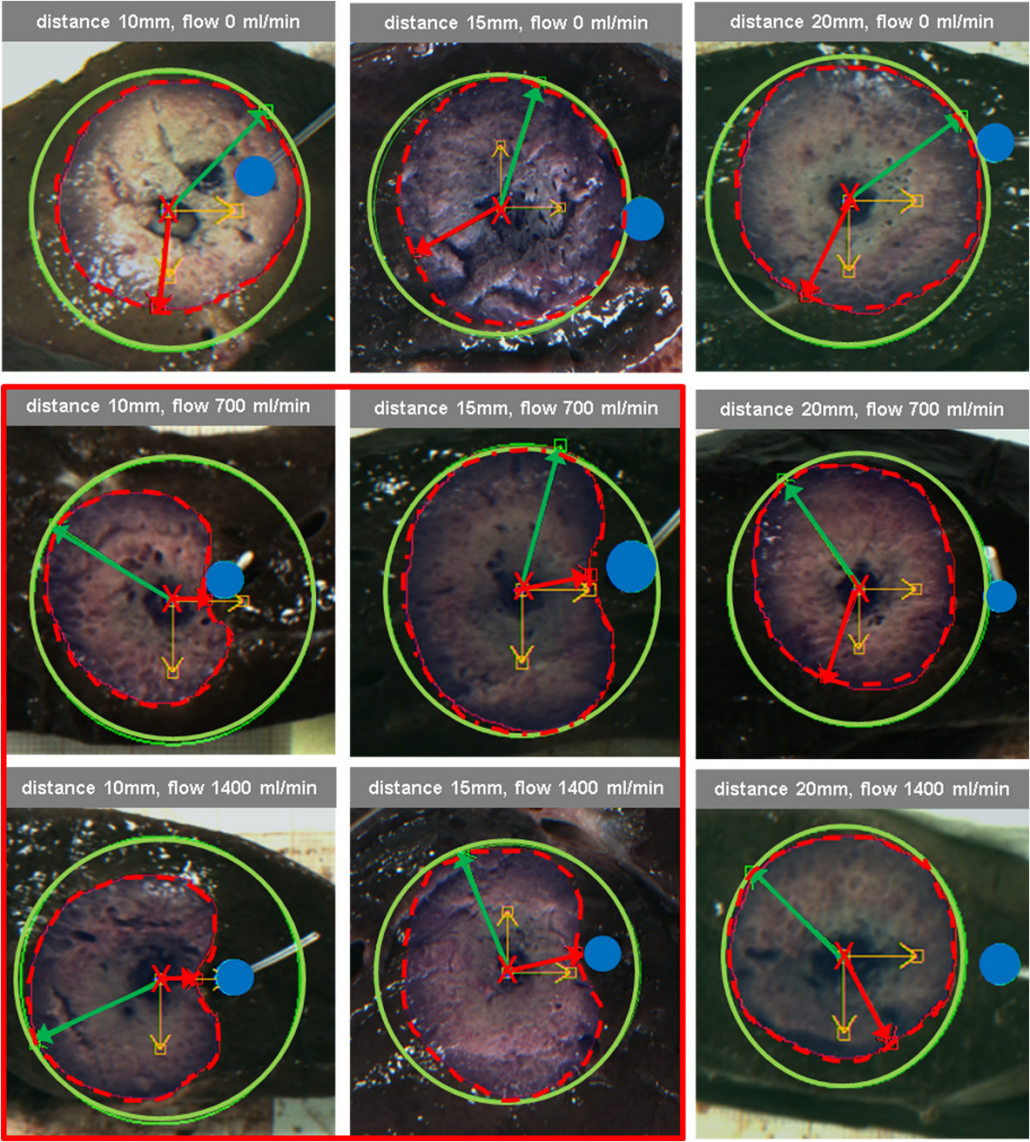
Demonstration of the heat sink effect in hepatic microwave ablation, depending on flow and distance to simulated hepatic vessels. Considerable changes in ablation zone morphology as reflected by minimum radius and lesion area can be observed in ablation procedures performed within 15mm of perfused vessels. Red cross = position of microwave antenna; blue circle = position of vessel; red arrow = minimal ablation zone radius; green arrow = maximum ablation zone radius; red dashed line = real ablation zone area; green line = idealized ablation zone area.

**Fig 2:**
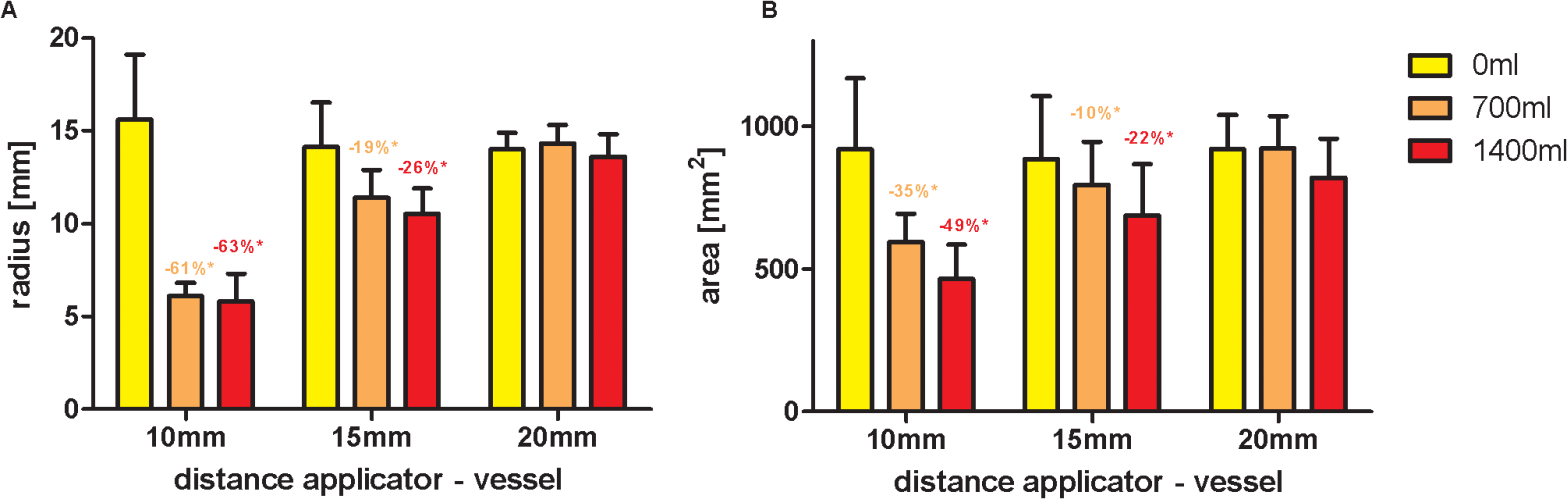
Minimum radius (A) and lesion area (B) of the ablation zone depending on applicator—vessel distance and vessel perfusion. In ablation procedures with vessels perfused with 700 ml/min located at 10 and 15mm distance, minimum radius of the resulting ablation zone was reduced by 61% and 19%, as compared to ablations with non-perfused vessels. Analogue, lesion area was reduced by 35% and 10%, respectively.

In ablation procedures with short antenna-vessel distance (10mm), ablation zones were significantly deformed based on different vessel perfusion parameters (p<0.05; detailed p-values are presented in Table 1). In non-perfused vessels minimum radius, maximum radius, total area and idealized lesion area of the ablation zone were 16mm, 21mm, 918mm^2^ and 1359mm^2^, respectively. In vessels perfused with 700 ml/min minimum radius, maximum radius, total area and idealized lesion area were 6mm, 19mm, 593mm^2^ and 1137mm^2^; in vessels perfused with 1400 ml/min respective values were 6mm, 16mm, 464mm^2^ and 817mm^2^. Thus, vessel perfusion with 700 and 1400 ml/min resulted in reduction of ablation zone area of 35% and 49%, respectively, as compared to ablation procedures with non-perfused vessels (p<0.0001) (Figs 1 and 2).

**Table 1:** Ablation zone parameters dependent on applicator–vessel distance and vessel perfusion. In addition, p-values for comparison of ablation zone parameters dependent on vessel flow are presented.

	flow	r_min_ [mm]	r_max_ [mm]	total area [mm^2^]	idealized area [mm^2^]
**Distance 10mm**		*p*<0.0001	*p* = 0.0022	*p*<0.0001	*p* = 0.0022
	*0 ml/min*	15 (12–25)	21 (16–29)	918 (614–1583)	1359 (835–2679)
	*700 ml/min*	6 (4–9)	19 (15–23)	593 (418–827)	1137 (707–1647)
	*1400 ml/min*	6 (5–7)	16 (13–20)	464 (329–671)	817 (530–1307)
**Distance 15mm**		*p* = 0.0005	*p* = 0.5407	*p* = 0.1531	*p* = 0.5407
	*0 ml/min*	14 (11–18)	19 (17–22)	885 (597–1305)	1160 (855–1576)
	*700 ml/min*	11 (10–15)	19 (17–24)	794 (576–1109)	1151 (876–1765)
	*1400 ml/min*	10 (8–13)	18 (13–22)	686 (361–922)	1033 (547–1452)
**Distance 20mm**		*p* = 0.1526	*p* = 0.2298	*p* = 0.1145	*p* = 0.2298
	*0 ml/min*	14 (13–15)	20 (16–23)	920 (655–1086)	1269 (784–1605)
	*700 ml/min*	13 (13–15)	21 (18–24)	922 (711–1137)	1355 (1018–1825)
	*1400 ml/min*	14 (12–15)	19 (16–24)	819 (569–1033)	1185 (794–1765)

In ablations with intermediate antenna-vessel distance (15mm) vessel perfusion resulted in reduction of minimum radius, maximum radius, total and idealized lesion area, as compared to ablations with non-perfused vessels. However, a statistical significant reduction was observed only regarding minimal ablation zone radius (p = 0.0005), which was 14mm, 11mm and 10mm at a flow of 0, 700 and 1400 ml/min, respectively. In ablation procedures with an antenna-vessel distance of 20mm, there was no significant effect of vessel perfusion on ablation zone morphology (p>0.05 for all parameters) (Table 1).

### Temperature

**Fig 3:**
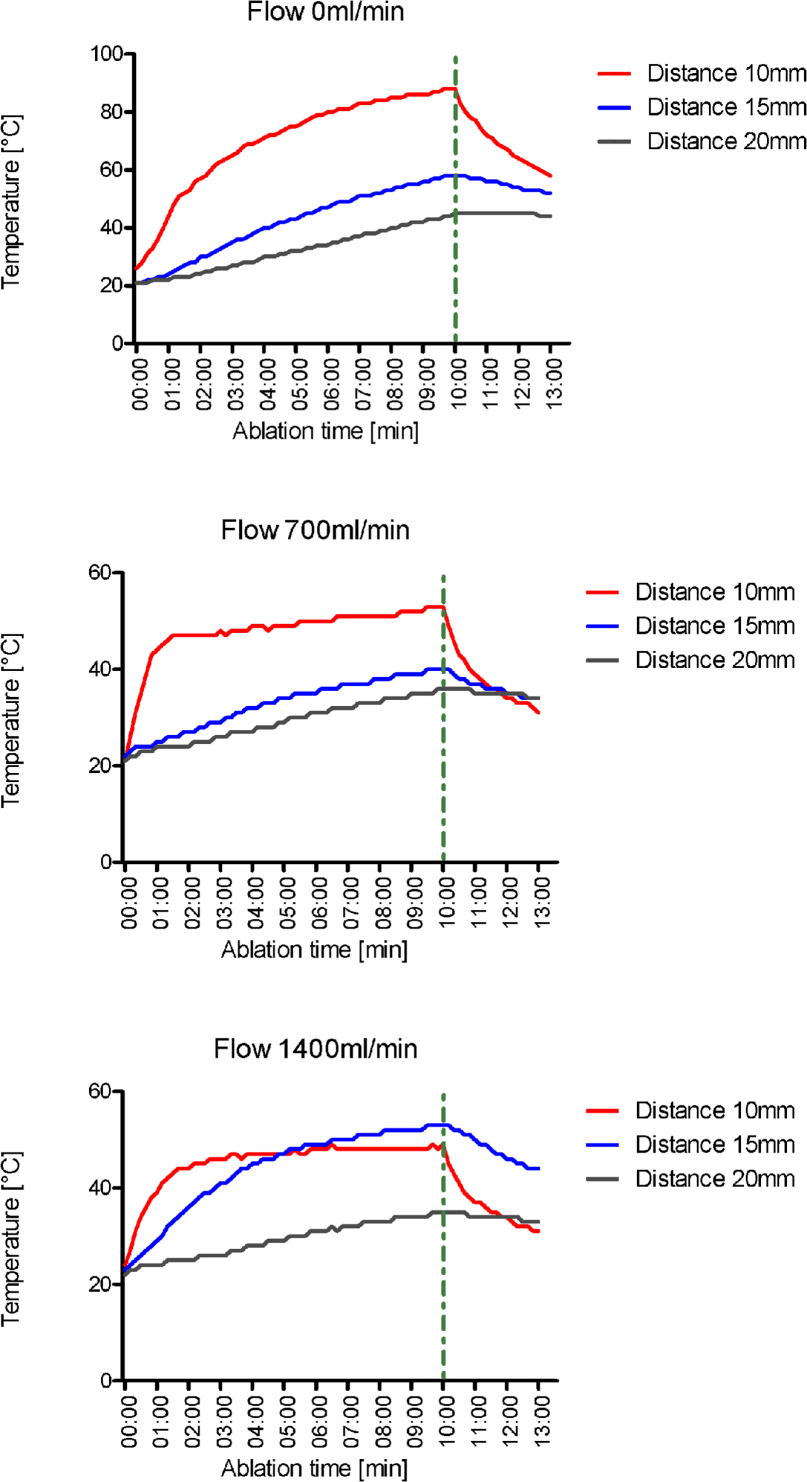
Temperature profile in ex microwave ablation of porcine liver, dependent on vessel perfusion and vessel–applicator distance. Maximum temperature during the ablation cycle decreased significantly with increasing distance between vessel and microwave antenna as well as with increasing vessel flow (p<0.05).

Maximum temperature during the ablation cycle decreased significantly with increasing distance between vessel and microwave antenna as well as with increasing vessel flow. At 10mm distance, maximum temperature at a flow of 0, 700 and 1400 ml/ min was 89°C, 54°C and 50°C, respectively (p<0.0001). At 15mm distance, maximum temperature was 58°C, 51°C and 43°C, respectively (p = 0.0006); at 20mm distance respective maximum temperatures were 45°C, 36°C and 35°C (p<0.0001) (Fig 3).

## Discussion

Incomplete tumor ablation due to heat sink effect caused by vessels located close to the target area is a known risk factor for local tumor recurrence in percutaneous thermoablation procedures [4]. Adjuvant techniques to occlude hepatic vascular inflow may be considered to minimize local tissue cooling, such as the Pringle maneuver, embolization [18] or the use of angioplasty balloons [5, 19, 20]. Technically, MWA has been propagated as the modality of choice over RFA when treating lesions located in close proximity to a sizeable vessel larger than 3mm in diameter [21].

In the present study the heat sink effect in hepatic microwave ablation was systematically assessed in an ex vivo porcine model. We investigated the effect of hepatic flow on lesion size, shape and volume as well as temperature varying both, the blood and the distance between vessel and microwave antenna. Considerable heat sink effect was observed in ablation procedures performed within the vicinity of perfused hepatic vessels. Local tissue cooling was not only dependent on distance to vessel, but also on flow rate. At a given flow rate, changes were more profound at closer vessel distance. Maximum temperature reached during the ablation cycle decreased significantly with increasing vessel flow. These findings are important when ablating liver lesions close to vessels, necessitating adjustments in antenna placement and treatment time in order to induce cell death.

Previous studies, using different and in large part less systematic approaches, have evaluated local tissue cooling effects in MWA. For example, Wright et al. made direct comparisons of ablation zones created by MWA and RFA [12]. The heat sink effect caused a 3.5 ± 5.3% deviation of the ablation zone in microwave ablated tissue compared with 26.2 ± 27.9% in RF ablated tissue (p<0.05). However, vessel size was not standardized and flow in the vessel was not assessed. Awad et al. performed microwave ablation in an in-vivo hepatic porcine model in peripheral and central locations, with and without total inflow obstruction [22]. For central ablations US-guided probe placement within 1cm of the origin of a major hepatic vein was performed. Vessel flow and exact distance were not further specified. Using an ablation system with a higher output (100W, 2450GHz) as compared to the one utilzed in our study, proximity and inflow occlusion did not significantly change lesion size or shape. Recently, Dodd and colleagues assessed the effect of variation of portal venous blood flow on radiofrequency and microwave ablations in a blood-perfused bovine liver model [10]. They performed sixty ablations ex vivo at 60W in 15 livers perfused via the portal vein at 60–100ml/min/100g. In contrast to our results, they concluded that the size of ablated lesions was not affected by changes in local blood flow. One important difference to our study, and a possible explanation for these diverging results may be the fact that large hepatic veins were purposely avoided in order to assess the effect of variation of global hepatic perfusion on ablation zone size. Thus the effect of local heat sink caused by adjacent large vessels on ablation zone size and configuration was not evaluated. In addition, flow rates was distinctly lower as compared to our study. The flow rates in our study were similar to those applied by Lehmann et al. for ex vivo quantification of the cooling effect of liver vessels on RFA [16], and primarily chosen to reflect portal venous and total hepatic blood flow, respectively [15].

Our study has several limitations. First, ablation procedures were performed at room temperature of 20°C ex-vivo in non-tumor bearing liver tissue. Even though in vivo, the threshold of 15mm (applicator vessel distance) observed in our model might be different, we were able to demonstrate that a heat sink effect in hepatic microwave ablation is does exist. However, our setup with glass tubes and temperature probes inserted into the liver would be challenging to perform in a living animal. Second, we did not directly compare the ablation zones created by MWA with those of RFA in our model. Also, we evaluated only one vessel size (diameter 5mm). As demonstrated previously by Yu et al. for microwave ablations performed within 1cm of hepatic veins, there is a correlation of the heat sink effect with vessel size [13]. At this point we can only speculate, that larger vessels (e.g. 7mm, 10mm) would have resulted in an even more pronounced heat sink effect. From a clinical perspective a systematic approach investigating the lower limits of vessel diameter, flow and distance at which significant tissue cooling is observed, would be meaningful. Given the high number of experiments, a prospective multicenter study is under way evaluating these parameters not only ex vivo, but also in an in vivo model.

In conclusion, based on this ex-vivo porcine model the size of the microwave ablation zone is effected by vessel distance and vessel flow rate. Even though MWA may be less affected by local tissue cooling as compared to RFA, significant heat sink effects need to be taken into account when ablating liver lesions close to vessels. Albeit the effects observed ex vivo in our model might be slightly different in vivo, adjustments of the ablation protocol (in terms of antenna placement and treatment time) seem to be necessary when treating patients with liver tumors.

## References

[1] Ahmed M, Brace CL, Lee FT, Goldberg SN. Principles of and advances in percutaneous ablation. Radiology. 2011;258(2): 351–359. 10.1148/radiol.10081634 21273519 PMC6939957

[2] Dupuy DE, Goldberg SN. Image-guided radiofrequency tumor ablation: challenges and opportunities-part II. J Vasc Interv Radiol. 2001;12(10): 1135–1148. 11585879 10.1016/s1051-0443(07)61670-4

[3] Hong K, Georgiades C. Percutaneous Tumor Ablation In: Hong K, Georgiades C (ed) Percutaneous Tumor Ablation. New York, Stuttgart: Thieme, 2011; p. 2–4.

[4] Lu DS, Raman SS, Limanond P, Aziz D, Economopu J, Busuttil R, et al Influence of large peritumoral vessels on outcome of radiofrequency ablation of liver tumors. J Vasc Interv Radiol. 2003;14(10): 1267–1274. 14551273 10.1097/01.rvi.0000092666.72261.6b

[5] Chinn SB, Lee FT, Kennedy GD, Chinn C, Johnson CD, Winter TC, et al Effect of vascular occlusion on radiofrequency ablation of the liver: results in a porcine model. AJR Am J Roentgenol. 2001;176(3): 789–795. 11222227 10.2214/ajr.176.3.1760789

[6] Kim YS, Rhim H, Cho OK, Koh BH, Kim Y. Intrahepatic recurrence after percutaneous radiofrequency ablation of hepatocellular carcinoma: analysis of the pattern and risk factors. Eur J Radiol. 2006;59(3): 432–441. 16690240 10.1016/j.ejrad.2006.03.007

[7] Al-Alem I, Pillai K, Akhter J, Chua TC, Morris DL. Heat Sink Phenomenon of Bipolar and Monopolar Radiofrequency Ablation Observed Using Polypropylene Tubes for Vessel Simulation. Surg Innov. 2013;21(3): 269–276. 10.1177/1553350613505713 24132470

[8] Schramm W, Yang D, Wood BJ, Rattay F, Haemmerich D. Contribution of direct heating, thermal conduction and perfusion during radiofrequency and microwave ablation. Open Biomed Eng J. 2007;1: 47–52. 10.2174/1874120700701010047 19662127 PMC2701080

[9] Skinner MG, Iizuka MN, Kolios MC, Sherar MD. A theoretical comparison of energy sources-microwave, ultrasound and laser-for interstitial thermal therapy. Phys Med Biol. 1998;43(12): 3535–3547. 9869030 10.1088/0031-9155/43/12/011

[10] Dodd GD, Dodd NA, Lanctot AC, Glueck DA. Effect of variation of portal venous blood flow on radiofrequency and microwave ablations in a blood-perfused bovine liver model. Radiology. 2013;267(1): 129–136. 10.1148/radiol.12120486 23297326

[11] Brannan JD, Ladtkow CM. Modeling bimodal vessel effects on radio and microwave frequency ablation zones. Conf Proc IEEE Eng Med Biol Soc. 2009; 5989–5992. 19965069 10.1109/IEMBS.2009.5334699

[12] Wright AS, Sampson LA, Warner TF, eMahvi DM, Lee FT. Radiofrequency versus microwave ablation in a hepatic porcine model. Radiology. 2005;236(1): 132–139. 15987969 10.1148/radiol.2361031249

[13] Yu NC, Raman SS, Kim YJ, Lassman C, Chang X, Lu DSK. Microwave liver ablation: influence of hepatic vein size on heat-sink effect in a porcine model. J Vasc Interv Radiol. 2008;19(7): 1087–1092. 10.1016/j.jvir.2008.03.023 18589324

[14] Bhardwaj N, Strickland AD, Ahmad F, El-Abassy M, Morgan B, Robertson GSM, et al Microwave ablation for unresectable hepatic tumours: clinical results using a novel microwave probe and generator. Eur J Surg Oncol. 2010;36(3): 264–268. 10.1016/j.ejso.2009.10.006 19880269

[15] Brown HS, Halliwell M, Qamar M, Read AE, Evans JM, Wells PNT. Measurement of normal portal venous blood flow by Doppler ultrasound. Gut. 1989;30(4): 503–509. 2653973 10.1136/gut.30.4.503 PMC1434022

[16] Lehmann KS, Ritz JP, Valdeig S, Knappe V, Schenk A, Weihusen A, et al Ex situ quantification of the cooling effect of liver vessels on radiofrequency ablation. Langenbecks Arch Surg. 2009;394(3): 475–481. 10.1007/s00423-009-0480-1 19274468

[17] Ritz JP, Lehmann KS, Isbert C, Reissfelder C, Albrecht T, Setin T, et al In-vivo evaluation of a novel bipolar radiofrequency device for interstitial thermotherapy of liver tumors during normal and interrupted hepatic perfusion. J Surg Res. 2006;133(2): 176–184. 16360176 10.1016/j.jss.2005.09.028

[18] Wacker FK, Reither K, Ritz JP, Roggan A, Germer CT, Wolf KJ. MR-guided interstitial laser-induced thermotherapy of hepatic metastasis combined with arterial blood flow reduction: technique and first clinical results in an open MR system. J Magn Reson Imaging. 2001;13(1): 31–36. 11169800 10.1002/1522-2586(200101)13:1<31::aid-jmri1005>3.0.co;2-i

[19] de Baere T, Bessoud B, Dromain C, Ducreux M, Boige V, Lassau N, et al Percutaneous radiofrequency ablation of hepatic tumors during temporary venous occlusion. AJR Am J Roentgenol. 2002;178(1):53–59. 11756087 10.2214/ajr.178.1.1780053

[20] Kobayashi A, Pulitano C. Treatment of huge HCC: extending the indications for liver resection. Ann Surg Oncol. 2008;15(5): 1549 10.1245/s10434-007-9773-1 18165876

[21] Carrafiello G, Lagana D, Mangini M, Fontana F, Dionigi G, Boni L, et al Microwave tumors ablation: principles, clinical applications and review of preliminary experiences. Int J Surg. 2008;6 Suppl 1:S65–69. 10.1016/j.ijsu.2008.12.028 19186116

[22] Awad MM, Devgan L, Kamel IR, Torbensen M, Choti MA. Microwave ablation in a hepatic porcine model: correlation of CT and histopathologic findings. HPB (Oxford). 2007;9(5):357–362. 18345319 10.1080/13651820701646222 PMC2225513

